# Extractions of steady-state auditory evoked fields in normal subjects and tinnitus patients using complementary ensemble empirical mode decomposition

**DOI:** 10.1186/s12938-015-0062-0

**Published:** 2015-07-26

**Authors:** Kuo-Wei Wang, Hsiao-Huang Chang, Chuan-Chih Hsu, Kuang-Chao Chen, Jen-Chuen Hsieh, Lieber Po-Hung Li, Po-Lei Lee, An-Suey Shiao

**Affiliations:** Department of Electrical Engineering, National Central University, No. 300, Jhongda Rd., Jhongli City, Taiwan; Department of Medical Imaging, Landseed Hospital, No. 77, Kuan-Tai Rd., Taoyuan, Taiwan; Cardiovascular Surgery Division, Surgery Department, Taipei Veterans General Hospital, No. 201, Sec. 2, Shipai Rd., Taipei, Taiwan; Division of Cardiovascular Surgery, Department of Surgery, Taipei Medical University Hospital, No. 252, Wu-Hsing St., Taipei, Taiwan; Department of Otolaryngology, Cheng-Hsin General Hospital, No. 45, Chenghsin St., Taipei, Taiwan; Institute of Brain Science, National Yang-Ming University, No. 155, Sec. 2, Linong St., Taipei, Taiwan; Center for Dynamical Biomarkers and Translational Medicine, National Central University, No. 300, Jhongda Rd., Jhongli, Taiwan; Department of Otolaryngology, Taipei Veterans General Hospital, No. 201, Sec. 2, Shipai Rd., Taipei, Taiwan

**Keywords:** Steady-state auditory evoked field, Magnetoencephalography, Complementary empirical mode decomposition

## Abstract

**Background:**

Auditory steady-state response (ASSR) induced by repetitive auditory stimulus is commonly used for audiometric testing. ASSR can be measured using electro-encephalography (EEG) and magnetoencephalography (MEG), referred to as steady-state auditory evoked potential (SSAEP) and steady-state auditory evoked field (SSAEF), respectively. However, the signal level of SSAEP and SSAEF are weak so that signal processing technique is required to increase its signal-to-noise ratio. In this study, a complementary ensemble empirical mode decomposition (CEEMD)-based approach is proposed in MEG study and the extraction of SSAEF has been demonstrated in normal subjects and tinnitus patients.

**Methods:**

The CEEMD utilizes noise assisted data analysis (NADA) approach by adding positive and negative noise to decompose MEG signals into complementary intrinsic mode functions (IMF). Ten subjects (five normal and five tinnitus patients) were studied. The auditory stimulus was designed as 1 kHz carrier frequency with 37 Hz modulation frequency. Two channels in the vicinities of right and left temporal areas were chosen as channel-of-interests (COI) and decomposed into IMFs. The spatial distribution of each IMF was correlated with a pair of left- and right-hemisphere spatial templates, designed from each subject’s N100m responses in pure-tone auditory stimulation. IMFs with spatial distributions highly correlated with spatial templates were identified using K-means and those SSAEF-related IMFs were used to reconstruct noise-suppressed SSAEFs.

**Results:**

The current strengths estimated from CEEMD processed SSAEF showed neural activities greater or comparable to those processed by conventional filtering method. Both the normal and tinnitus groups showed the phenomenon of right-hemisphere dominance. The mean current strengths of auditory-induced neural activities in tinnitus group were larger than the normal group.

**Conclusions:**

The present study proposes an effective method for SSAEF extraction. The enhanced SSAEF in tinnitus group echoes the decreased inhibition in tinnitus’s central auditory structures as reported in previous studies.

## Background

Auditory steady-state responses (ASSR) are the net effect of entrained background activity and overlaid cortical evoked responses [1]. ASSR is a sinusoidal electrical or magnetic response in cortex induced by periodically presented auditory stimulus which has been regarded as a potential tool to provide valuable diagnostic information, especially for hearing loss [2, 3], audiometric hearing assessment [4, 5], and evaluation of aural rehabilitation [6]. ASSR can be induced by either amplitude modulation (AM) [7–9] or frequency modulation (FM) technique [10]. It has been demonstrated that both the carrier frequency and modulation method of auditory stimulus can affect the values of induced ASSR [11]. Hart et al. used fMRI and found AM stimulus can induce higher auditory activities than FM stimulus [12]. Though FM stimulus is supposed to be more related to human speech recognition and is essential for accurate phoneme recognition in speech [13], the FM stimulus is seldom used for clinical use, due to the lack of intuitive understanding of FM processing, lack of simple equipment to generate stimulus, and lack of solid evidence for its role in speech processing [14]. Therefore, AM stimulus was chosen to induce ASSR in this study.

Magnetoencephalography (MEG) has the salient feature of high temporal resolution superior to other brain imaging modalities (e.g., positron emission tomography (PET), fMRI, etc.). Compared to PET and fMRI studies, the PET and fMRI signals are recorded on a time-scale in seconds and thus are unable to resolve the neural dynamics of basic sound processing [15–17]. In addition, the MEG measures the neuromagnetic field produced by neuroelectrical currents in human brain. The measured neuromagnetic field is less distorted by skull and scalp which results in better source localization compared to EEG. The MEG is therefore chosen as a powerful tool to study auditory evoked responses.

Ross et al. utilized MEG to measure steady-state auditory field (SSAEF) induced by 40 Hz AM auditory stimulus, and proposed the SSAEF might be available to probe profound sound processing in central nerve system (CNS). Plurde and Picton found suppressed SSAEF during sedation and anesthesia [18]. Cohen et al. found attenuated SSAEF during sleeping states [19]. Draganova et al. utilized diotic and dichotic stimulation to produce peripheral and central beta responses, respectively [20]. Diesch et al. studied enhanced SSAEF in tinnitus patients compared to normal group [21]. According to the aforementioned studies, though the SSAEF has been proposed as a potential tool, however, the weak signal level of SSAEF makes it susceptible to noise.

Empirical mode decomposition (EMD)-based methods, i.e., EMD, ensemble empirical mode decomposition (EEMD) and complementary ensemble empirical mode decomposition (CEEMD) [22–25], have been used to extract nonstationary signals in many applications, such as analysis of blood pressure [26], detection of heart-rate variability in electrocardiogram (ECG) [27, 28], pulmonary hypertension [26], brain computer interface [29, 30], and etc. The present study adopted CEEMD to extract SSAEF. Comparing CEEMD with EMD and EEMD, the CEEMD can avoid mode-mixing problem in conventional EMD method and achieve smaller residual noise than EEMD. This study demonstrated the effectiveness of CEEMD in extraction of SSAEF.

## Methods

### Auditory stimulation

The steady-state auditory stimulus was created by 1 kHz carrier sinusoidal wave and modulated at 37 Hz with 100% modulation depth [31]. Monaural auditory stimulus was presented to right and left ears for each participant in separate sessions. The steady-state auditory stimulus was 180-s duration for each session, and triggers were given at every second. In addition to steady-state auditory stimulus, 100 trials of short pure-tone bursts (1 kHz carrier frequency with 300 ms duration) were applied binaurally to induce auditory evoked fields (AEF) for the generation of spatial template (see below). The sound pressure of auditory stimulus was set at 75 dB (SPL) which was generated by digital-to-analog conversion card (D/A) conversion card (NI USB-6259, National Instrument, Austin, Texas, USA) controlled by LabView software (National Instruments, USA).

### Subjects and tasks

Five normal subjects, numbered as N1–N5 (four males, one female, all right handed; mean age 52.2 ± 6.9 years, ranged from 45 to 62 years) and five tinnitus patients, numbered as P1 to P5 (four males, one female, all right handed; mean age 57.8 ± 8.8 years, ranged from 48 to 69 years), were recruited to participate in this experiments. Among the tinnitus patients, three patients were right-ear tinnitus and the other two were left-ear tinnitus patients. Subjects were asked to sit in a comfortable armchair in a dimly illuminated electro-magnetic shielded room. All participants were requested to participate in three auditory stimulation sessions, including one binaural pure-tone stimulation and two monaural steady-state auditory stimulations (one for right ear and one for left ear). Three-minute empty room measurement was also recorded for each participant to monitor MEG background noise. All participants gave informed consent, and the study was approved by the Ethics Committee of Institutional Review Board (IRB), Taipei Veterans General Hospital, Taiwan. The demographic data of tinnitus patients is provided in Table 1.

**Table 1:** Demographic data of tinnitus patient

Patient #	Gender	Age (years)	Duration (years)	Perceived tinnitus location
P1	M	50	5	Right ear
P2	M	48	6	Left ear
P3	M	64	5	Left ear
P4	F	69	3	Both ears
P5	M	59	3	Right ear

### MEG recordings

Cortical magnetic signals were recorded with a 306-channel (102 sensor units) whole-head neuromagnetometer (band-pass filtered within 0.05–250 Hz; digitized at 1 kHz; Vectorview; Neuromag Ltd., Helsinki, Finland) with subjects in sitting position. Each sensor unit was composed of a pair of planar gradiometers and a magnetometer. The magnetometer measured magnetic flux ($B_{z}$), normal to the sensor unit, while the gradiometers measured two tangential derivatives of $B_{z}$ ($\partial B_{z} /\partial x$ and $\partial B_{z} /\partial y$, mutually orthogonal) along the longitudinal and latitudinal directions, respectively. Bipolar horizontal and vertical electro-oculograms (EOG) were recorded using electrodes placed below and above the left eye and at the bilateral outer canthi to monitor eye movement and blinks. The exact position of the head with respect to the sensor array was determined by measuring magnetic signals from four head position indicator (HPI) coils placed on the scalp. Coil positions were identified with a three-dimensional digitizer with respect to three predetermined landmarks (naison and bilateral preauricular points) on the scalp, and this data was used to superimpose MEG source signals on individual MRI images obtained by a 3.0 T Bruker MedSpec S300 system (Bruker, Kalsrube, Germany). The anatomical image was acquired using a high-resolution T1-weighted, 3D gradient-echo pulse sequence (MDEFT: modified driven equilibrium Fourier transform; TR/TE/TI = 88.1 ms/4.12 ms/650 ms, 128 × 128 × 128 matrix, FOV = 250 mm).

### Complementary empirical mode decomposition (CEEMD) and creation of spatial maps for intrinsic mode functions

The whole-head MEG signals recorded in monaural steady-state auditory stimulations were stored in hard disk for subsequent off-line CEEMD processing. Since the planar gradiometers have better sensitivity and localized power [9, 32], only MEG gradiometers were used for data analysis in this study [33, 34]. The two gradiometer channels, one at right hemisphere and one at left hemisphere in the vicinity of auditory areas, presenting largest AEFs, were designated as right and left channel-of-interest (COI) channels. The signals (180 s in each session) recorded from the two COIs located in both hemispheres were separately processed by CEEMD to extract noise-suppressed SSAEF.

For each session, the MEG data set contained *K* sensor units (*K* = 102) with *2* *K* gradiometer channels and *N* (*N* = 1,80,000) time points. The paired gradiometers ($\partial B_{z} /\partial x$ and $\partial B_{z} /\partial y$, along the longitudinal and latitudinal directions), were arranged into two *K* × *N* submatrices, **B**_*x*_ and **B**_*y*_. The data matrix **B** was arranged as $${\mathbf{B}}_{2K \times N} = \left[ {\begin{array}{*{20}c} {{\mathbf{B}}_{x}^{T} } & {{\mathbf{B}}_{y}^{T} } \\ \end{array} } \right]^{T}$$.

The CEEMD adopts the idea of noise-assisted data analysis (NADA) by adding positive and negative white noise in pairs to generate complementary IMFs. Each IMF of CEEMD is the ensemble average of complementary IMFs in the same scale. For one MEG gradiometer recording $\vec{z}$, the signal was decomposed by the following CEEMD steps [25]:

1. Set an ensemble number *EN* (*EN* = 1,000) for the CEEMD process;
2. Set $\vec{x}(i) = \vec{z}$ (*i* = 1 at the beginning of the CEEMD process), where $\vec{z}$ is an MEG epoch of $1 \times N$ vector with *N* sampled points.
3. Generate white noise, $\vec{w}$;
4. Add white noise $\vec{w}$ to $\vec{x}(i)$ [signal-to-noise ratio (SNR) = 0.01] to obtain a pair of noise-added data $\vec{x}_{p} (i)$ and $\vec{x}_{n} (i)$, in which $\vec{x}_{p} (i) = \vec{x}(i) + \vec{w}$ and $\vec{x}_{n} (i) = \vec{x}(i) - \vec{w}$, respectively;
5. Apply EMD to $\vec{x}_{p} (i)$ and $\vec{x}_{n} (i)$, separately, to generate two series of IMFs ${\mathbf{C}}_{p} (i)$ and ${\mathbf{C}}_{n} (i)$, where ${\mathbf{C}}_{p} (i)$ and ${\mathbf{C}}_{n} (i)$ are two $J \times N$ matrix, containing *J* IMFs obtained from $\vec{x}_{p} (i)$ and $\vec{x}_{n} (i)$, respectively;
6. Repeat step (2) to step (5) until *i* reaches the preset ensemble number *EN*;
7. Calculate the ensemble means of IMFs ${\mathbf{C}}_{{}} = \frac{1}{2 \cdot EN}\sum\limits_{i = 1}^{EN} {{\mathbf{C}}_{p} (i) + {\mathbf{C}}_{n} (i)} = \left[ {\vec{c}_{1}^{\text{T}} \, \vec{c}_{2}^{\text{T}} \, \cdots \, \vec{c}_{J}^{\text{T}} } \right]^{\text{T}}$, where $\vec{c}_{i}$ is the *i*^th^ IMF of CEEMD.

In this study, we identified SSAEF-related IMFs based on a template-based matching approach. A spatial map was created for each IMF in order to facilitate the IMF selection process. The correlation coefficients between each IMF and the measured signals in all MEG gradiometers were computed. All the correlation coefficient values were then used to create the spatial map by multiplying the data matrix **B** with **C**^**T**^:

1 $${\mathbf{M}} = {\mathbf{B}} \cdot {\mathbf{C}}^{{\mathbf{T}}} = \left[ {{\mathbf{M}}_{x}^{T} \,{\mathbf{M}}_{y}^{T} } \right]^{\rm T} ,$$

where ${\mathbf{M}}_{x}$ represents the correlation values between the IMFs and the data of longitudinal gradiometers, and ${\mathbf{M}}_{x}$ represents the correlation values between the IMFs and the data of latitudinal gradiometers, respectively.

Spatial map for *j*^th^ IMF can then be created by

2 $$\vec{S}_{j} = \left[ {\begin{array}{*{20}c} {\sqrt {m_{x} (1,j)^{2} + m_{y} (1,j)^{2} } } & \cdots & {\sqrt {m_{x} (\tfrac{M}{2},j)^{2} + m_{y} (\tfrac{M}{2},j)^{2} } } \\ \end{array} } \right] ,$$

where $$\overset{\lower0.5em\hbox{$\smash{\scriptscriptstyle\rightharpoonup}$}} {S}_{j}$$ contains the vector sums of the correlation values in the *j*^th^ column vector of **M**, which presents the topographic distribution of *j*^th^ IMF over all MEG sensors.

### Selection of pertinent IMFs using K-means for reconstruction of noise-suppressed SSAEF

Since different brain areas usually have their own specialized functions, the spatial distribution, rather than temporal waveform, was utilized for selecting SSAEF-related IMFs. Accordingly, the correlation coefficient between the spatial map of each IMF and the spatial template (see below) was calculated. The correlation coefficients obtained from all IMFs were further categorized into highly-, middlely-, and lowly-correlated groups using K-means classifier [35]. Only those IMFs belonged to highly-correlated group are chosen as SSAEF-related IMFs and subjected to the subsequent reconstruction of noise-suppressed SSAEF. The reconstruction process was achieved by summating the chosen IMF portions in all MEG channels as:

3 $${\mathbf{B}}_{{{\mathbf{recon}}}} = \sum\limits_{{i \in S_{1} }} {} \frac{{{\mathbf{B}} \cdot \vec{c}_{i}^{T} }}{{\left\| {\vec{c}_{i} } \right\|^{2} }} \cdot \vec{c}_{i} ,$$

where $S_{1}$ is a group contains the index number of the IMFs belonged to highly-correlated group and **B**_**recon**_ is a *2K* × *N* matrix, in which the first *K* rows contain the reconstructed data of longitudinal gradiometers and the other *K* rows contain the reconstructed data of latitudinal gradiometers. The reconstructed magnetic fields **B**_**recon**_ were further filtered within 1–100 Hz to remove high-frequency spiky noise.

The auditory-induced source activities were estimated by means of minimum norm estimation (MNE) (BrainStorm software, University of South California; http://neuroimage.usc.edu/brainstorm), with realistic head model generated from individual magnetic resonance image (MRI) using brainVISA software (http://brainvisa.info/). The estimated neural sources were overlaid on anatomical MRI and only those cortical surface nodes with source amplitudes survived statistical significance (p < 0.05) among total surface nodes were rendered on MRI.

### Creation of right- and left-hemisphere spatial templates based on amplitude of N100m peak in auditory evoked fields (AEF)

The present CEEMD-based approach utilized a template matching process. Spatial maps of IMFs were correlated with the pre-defined templates to identify auditory-related IMFs. The N100m peak in AEF has been treated analogous to N100 (or N1) peak in EEG auditory evoked potential (AEP) [36]. Both are generated from the neural populations in the primary and association auditory cortices, located in the superior temporal gyrus [37].

**Figure 1:**
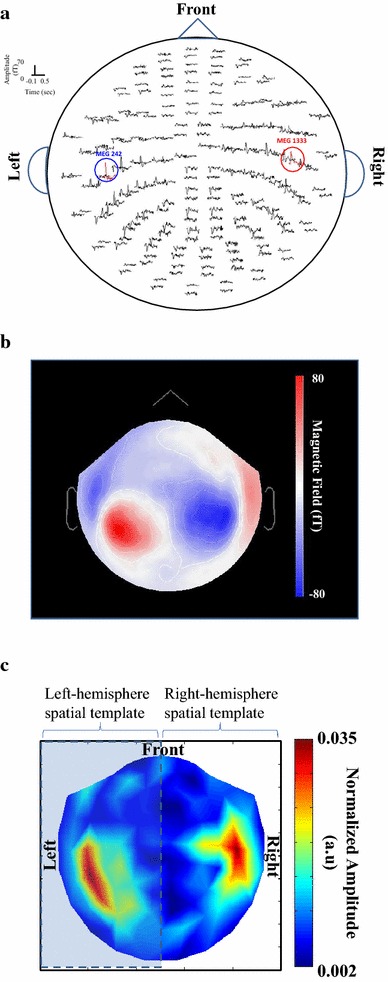
Spatial template creation using N100m. **a** The channel plot of induced AEF in subject I. **b** The magnetic fields of N100m in all MEG channels. **c** The normalized map of absolute value of **b**. **c** is separated into right (*unshaded*) and left (*shaded*) hemispheric parts for selecting SSAEF-related IMFs.

Since SSAEF is also the neuromagnetic response originated from auditory cortex, the AEF obtained from binaural pure-tone stimulation in each subject was used to create his/her own right- and left-hemisphere spatial templates, in order to facilitate the selection of SSAEF-related IMFs. Figure 1a shows the channel plot of AEF in binaural pure-tone stimulation in subject I. It can be observed that the MEG1333 (marked by red circle) and MEG242 (marked by blue circle) which showed largest N100m peaks in the right and left hemispheres, respectively, were designated as COIs. The magnetic fields at the latency of N100m are shown in Figure 1b and its absolute value is shown in Figure 1c. The Figure 1c was then divided into right- (unshaded part) and left-hemisphere (shaded part) spatial templates to facilitate the IMF selections in right and left hemispheres, respectively.

**Figure 2:**
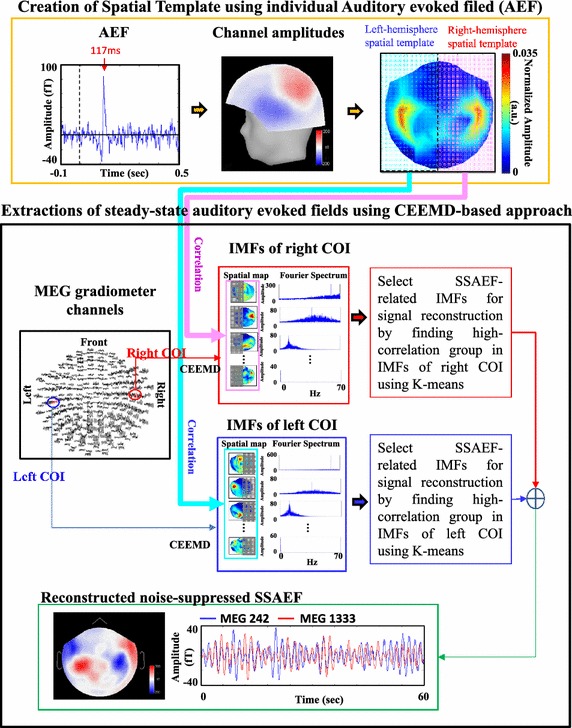
The schematic diagram for the signal processing of the proposed CEEMD-based approach.

Figure 2 shows the schematic diagram for the signal processing of the proposed CEEMD-based approach. The MEG signals recorded from right and left COIs (MEG 1333 and MEG 242) were decomposed separately into different sets of IMFs. The right/left parts of spatial maps generated from IMFs of right/left COI were correlated with right-/left-hemisphere spatial template to find SSAEF-related IMFs. The signals reconstructed from SSAEF-related IMFs of right and left COIs were summated to obtain noise-suppressed SSAEFs.

### Calculation of laterality index (LI)

Because the laterality index (LI) has been introduced as an effective indicator to quantitatively describe the ASSR hemispheric asymmetry [16], the right hemispheric laterality of auditory-induced source activity was calculated in this study. The LI was calculated as the difference of source activities in right and left auditory areas, normalized to the current strength of their summation. The calculation of LI is represented as follows,

4 $$LI = (E\{ I_{Right} \} - E\{ I_{left} \} )/(E\{ I_{right} \} + E\{ I_{left} \} ),$$

in which $E\left\{ { \, \cdot \, } \right\}$ is the operator of expected value, and *I*_*right*_ and *I*_*left*_ are the estimated auditory-induced source activities in right and left auditory areas, respectively.

## Results

**Figure 3:**
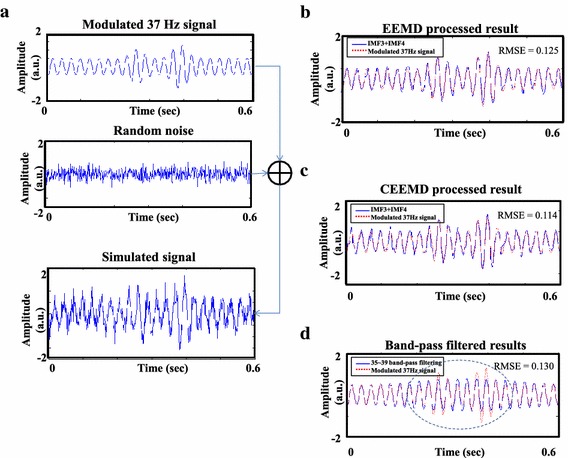
Demonstration of signal extraction in a simulated 37 Hz SSAEF using EEMD, CEEMD and band-pass filtering. **a** The simulation of a modulated 37 Hz SSAEF with −1 dB SNR. **b**, **c** The SSAEFs reconstructed by the summation of IMF2 and IMF3 using EEMD and CEEMD with ensemble number equal to 1,000 times, respectively. **d** The SSAEF extracted by 35–39 Hz band-pass filtering.

**Figure 4:**
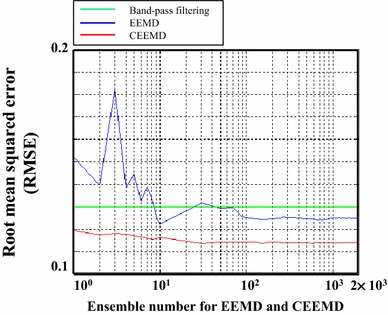
The RMSE values of extracted SSAEF in Figure 3 with different ensemble numbers.

To demonstrate the capability of CEEMD in the reduction of residual noise, a modulated 37 Hz SSAEF signal with random noise was simulated (see Figure 3a). Figure 3b–d show the results of SSAEFs extracted by EEMD, CEEMD and 35–39 Hz band-pass filtering, in which the ensemble numbers of EEMD and CEEMD were both 1,000. The root-mean-square errors (RMSE) for EEMD, CEEMD and band-pass filtering were 0.125, 0.114 and 0.130, respectively. In Figure 3d, the SSAEF extracted by band-pass filtering was distorted (blue line). The two modulated envelope peaks were smeared as one peak (dashed line). It indicates inappropriate setting of filter parameter might result in wrong interpretation. To compare the performance of EEMD and CEEMD, RMSEs of the simulation example with different ensemble number (from 1 to 2,000) were calculated and plotted in Figure 4. The RMSEs of CEEMD with different ensemble number were all smaller than those processed by EEMD.

**Figure 5:**
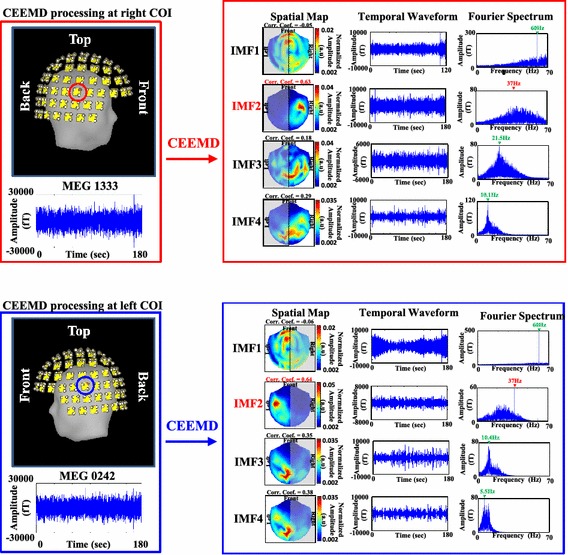
The spatial maps, IMF waveforms and Fourier spectra of the first four IMFs decomposed from right- and left-hemisphere COIs. The IMF2s decomposed from right and left COIs (correlation coefficient = 0.63 and 0.64 in right and left COI decompositions, respectively) were chosen for signal reconstruction.

This study took the advantage of CEEMD in extracting nonstationary signal [25]. Figure 5 shows the IMFs decomposed by CEEMD in subject I. The upper panels (marked by red rectangle) show the decomposition of IMFs by applying CEEMD at right COI (MEG 1333, marked by red circle), and the lower panels (marked by blue rectangle) show the IMFs decomposed from left COI (MEG242, marked by blue circle). The spatial maps, temporal waveforms and Fourier spectra of the IMFs were shown in the first, second and third columns, respectively. At right COI (upper panels), the peak frequencies in IMF1, IMF2, IMF3, and IMF4 were 60, 37, 21.5, and 10.1 Hz, representing the 60 Hz electricity noise, 37 Hz SSAEF signal, beta rhythm, and alpha rhythm, respectively. The values of correlation coefficients between right-hemisphere spatial template and right parts of spatial maps were 0.05, 0.63, 0.18, and 0.29, respectively. The IMF2 manifested a high correlation value centered at right temporal area was chosen as SSAEF-related IMF. At left COI (lower panels), the IMF1, IMF2, IMF3 and IMF4 had peak frequencies at 60, 37, 10.4, and 5.5 Hz, representing the 60 Hz electricity noise, 37 Hz SSAEF signal, alpha rhythm, and low-frequency noise, respectively. The calculated correlation coefficients between left-hemisphere spatial template and left parts of spatial maps were 0.06, 0.64, 0.35, and 0.38, respectively. Both the IMF2s obtained from right and left COIs, classified as highly-correlated group using K-means (correlation coefficients marked in red), were then chosen as SSAEF-related IMFs to reconstruct whole-head SSAEF-related oscillatory activities using Eq. (3).

**Figure 6:**
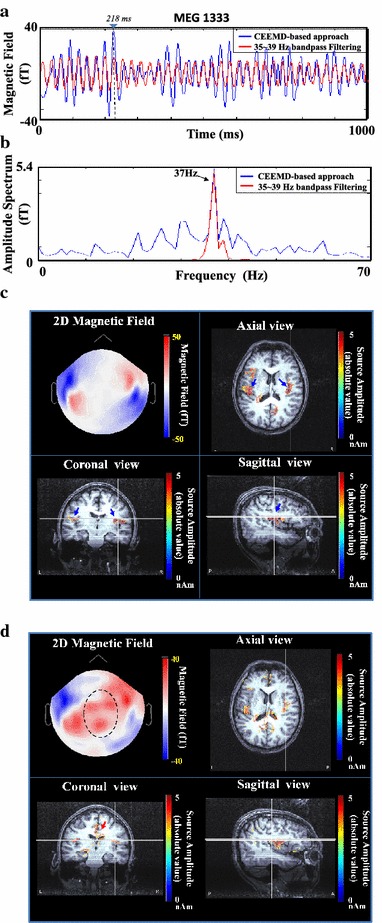
The comparison of SSAEFs extracted by our CEEMD-based approach and conventional filtering method in subject I. **a** One example of one-second SSAEF waveforms obtained from the two methods. **b** The Fourier spectra of **a**. **c** The spatial map and current sources of CEEMD processed SSAEF estimated at 218 ms in **a**. **d** The spatial map and current sources of SSAEF processed by 35–39 Hz bandpass filtering at 218 ms in **a**.

To demonstrate the effectiveness of CEEMD in SSAEF extraction, conventional filtering method which filtered whole-head MEG signals within 35–39 Hz (zero-phase fourth-ordered Butterworth filter) was also applied for comparison purpose. Figures 6 shows the SSAEFs processed by our CEEMD-based method and conventional filtering method. Figure 6a, b present the temporal waveforms and Fourier spectra of one-second MEG activities at MEG1333 channel which were processed by CEEMD-based approach (marked in blue) and conventional filtering method (marked in red), respectively. It can be observed that both the MEG signals processed by CEEMD and conventional filtering method preserved similar signal phase. No conspicuous phase difference or phase distortion between these two signals was observed. In addition, the CEEMD-based approach preserved fast changes of time-varying features in signal waveform (blue line in Figure 6a) with broadened 37 Hz spectral peak (blue line in Figure 6b). In contrast, fixed parameters for band-pass filtering could result in over-filtered MEG signal with smeared signal features (red line in Figure 6a). Figures 6c and 6d show the results of source estimations of SSAEF at the latency of 218 ms (marked in Figure 6a) processed by CEEMD approach and 35–39 Hz bandpass filtering, respectively. In Figure 6c, two clear influx-outflux fields were observed with neural activations concentrated at superior temporal regions in both hemispheres (marked by blue arrows). Compared to the magnetic fields processed by conventional filtering method in Figure 6d, interference was observed in the center region (marked by dotted circle) and resulted in the artifact of source activations at midline areas (marked by red arrows), which might be caused by the inevitable incursion of 37 Hz components from SSAEF-unrelated noise.

In our study, source activities were estimated by MNE using Brainstorm software (University of South California). The current strengths in auditory cortex were estimated from each session (180 s long) and the absolute values of current strengths were calculated for statistical analyses. Tables 2 and 3 list the results of estimated current strengths in normal subjects and tinnitus patients, respectively. It can be observed that the current strengths in most CEEMD processed data were significantly larger than those processed by the conventional filtering method (paired *t* test).

**Table 2:** Comparison of estimated current strengths in normal subjects

Normal subject	The proposed CEEMD approach				Traditional filtering method			
	Current strength of left ear stimulation		Current strength of right ear stimulation		Current strength of left ear stimulation		Current strength of right ear stimulation	
	Left auditory cortex (nAm)	Right auditory cortex (nAm)	Left auditory cortex (nAm)	Right auditory cortex (nAm)	Left auditory cortex (nAm)	Right auditory cortex (nAm)	Left auditory cortex (nAm)	Right auditory cortex (nAm)
N1	2.64 ± 0.93**	5.32 ± 1.40*	2.16 ± 0.96*	2.79 ± 1.36	2.16 ± 0.73	5.14 ± 1.26	2.05 ± 0.57	2.70 ± 1.26
N2	2.59 ± 0.80**	3.34 ± 1.02**	2.00 ± 0.82**	2.86 ± 1.06**	1.47 ± 0.70	2.11 ± 0.64	1.54 ± 0.78	1.74 ± 0.65
N3	2.82 ± 1.00**	5.44 ± 1.70*	4.58 ± 1.34**	4.15 ± 1.66**	2.61 ± 1.01	5.04 ± 1.57	4.12 ± 1.15	3.36 ± 1.34
N4	3.78 ± 1.33**	8.66 ± 1.64**	4.97 ± 1.66**	6.29 ± 1.41**	2.37 ± 0.99	5.24 ± 1.18	4.01 ± 1.23	5.29 ± 1.19
N5	3.09 ± 1.15**	5.77 ± 1.17**	2.87 ± 0.80**	6.02 ± 1.75**	2.22 ± 0.76	4.33 ± 0.88	2.14 ± 0.62	4.27 ± 1.24
Mean ± std	2.98 ± 1.11	5.68 ± 2.22	3.28 ± 1.66	4.39 ± 2.14	2.19 ± 1.07	4.33 ± 1.64	2.76 ± 1.40	3.39 ± 1.65

The current strengths of CEEMD processing greater than traditional filtering method.

* p < 0.05 by paired t test.

** p<0.01 by paired t test.

**Table 3:** Comparison of estimated current strengths in tinnitus patients

Tinnitus patient	The proposed CEEMD approach				Traditional filtering method			
	Current strength of left ear stimulation		Current strength of right ear stimulation		Current strength of left ear stimulation		Current strength of right ear stimulation	
	Left auditory cortex (nAm)	Right auditory cortex (nAm)	Left auditory cortex (nAm)	Right auditory cortex (nAm)	Left auditory cortex (nAm)	Right auditory cortex (nAm)	Left auditory cortex (nAm)	Right auditory cortex (nAm)
P1	3.12 ± 0.98**	6.15 ± 1.62**	3.45 ± 1.15	4.21 ± 1.60**	2.5 ± 0.78	4.98 ± 1.31	3.68 ± 1.95	3.63 ± 1.38
P2	3.87 ± 1.60*	6.25 ± 1.70**	3.65 ± 1.48**	8.83 ± 2.12**	3.52 ± 1.15	4.93 ± 1.34	3.01 ± 1.10	6.00 ± 1.44
P3	3.38 ± 1.36**	4.66 ± 1.47**	5.07 ± 2.10**	10.28 ± 3.88**	2.03 ± 0.69	3.23 ± 1.02	2.75 ± 1.66	7.07 ± 2.64
P4	3.93 ± 1.09**	8.87 ± 1.41**	4.68 ± 1.77**	2.86 ± 1.16**	3.16 ± 0.63	6.54 ± 1.04	3.06 ± 1.28	2.11 ± 0.86
P5	4.28 ± 1.51**	5.70 ± 1.52**	4.05 ± 1.49**	7.90 ± 2.53**	3.09 ± 1.03	3.61 ± 0.96	2.57 ± 1.05	4.37 ± 1.40
Mean ± std	3.76 ± 1.39	6.30 ± 2.06	4.14 ± 1.74	6.92 ± 3.68	2.86 ± 0.86	4.66 ± 1.13	2.98 ± 1.53	4.67 ± 2.33

The current strengths of CEEMD processing greater than traditional filtering method.

* p < 0.05 by paired t test.

** p < 0.01 by paired t test.

Comparing the current strengths of CEEMD-based approach in right and left auditory areas, the current strengths in right-hemisphere auditory cortex were larger than those in left hemispheres in normal subjects (4.39 ± 2.14 vs 3.28 ± 1.66 nAm in right-ear stimulation; 5.68 ± 2.22 vs 2.98 ± 1.11 nAm in left-ear stimulation) (see Table 2) and in tinnitus patients (6.92 ± 3.68 vs 4.14 ± 1.74 nAm in right-ear stimulation; 6.30 ± 2.06 vs 3.76 ± 1.39 nAm in left-ear stimulation) (see Table 3). The observation echoed the right hemispheric laterality of ASSR proposed by Ross et al. [16]. In addition, the current strengths of SSAEF in tinnitus patients were larger than those in normal subjects which indicate decreased inhibition in central auditory structures [38].

**Figure 7:**
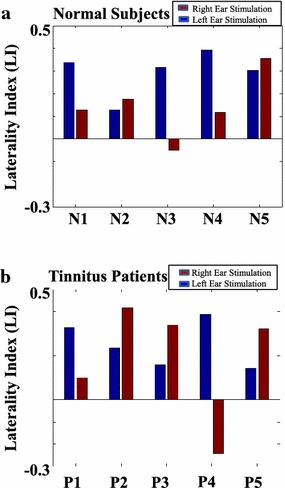
The LI values during right and left auditory stimulations in normal subjects and tinnitus patients. **a** The LI values calculated from right-ear (*red bars*) and left-ear (*blue bars*) stimulations in normal subjects. **b** The LI values calculated from right-ear (*red bars*) and left-ear (*blue bars*) stimulations in tinnitus patients.

Figure 7 shows the LI values estimated at source domain during right and left auditory stimulations in all subjects. In normal subjects, the LI values were 0.13, 0.18, −0.05, 0.12, and 0.35 for N1–N5 in right-ear stimulations (red bars in Figure 7a), and the LI values were 0.34, 0.13, 0.32, 0.39, and 0.30 for N1–N5 in left-ear stimulation (blue bars in Figure 7a), respectively. In tinnitus patients, the LI values were 0.10, 0.42, 0.34, −0.24, and 0.32 for P1–P5 in right-ear stimulations (red bars in Figure 7b), and the LI values were 0.33, 0.24, 0.16, 0.39, and 0.14 for P1–P5 in left-ear stimulations (blue bars in Figure 7b), respectively. Except the right-ear stimulation in N3 and P4, all subjects had right-hemisphere dominance (LI ≥ 0) of SSAEF during steady-state auditory stimulations.

## Discussions

Human ASSR has been reported as an effective tool for the measurements of hearing loss in adults and children [39, 40], Anesthesia level [41], and tinnitus [38, 42]. Since the SNR in ASSR signal is weak [43], it usually requires average over a large amount of epochs for noise suppression [44]. Traditional averaging techniques presume the measured auditory responses are stationary and the segmented data are identical. Nevertheless, subject’s performance can be varied owing to fluctuations in subject’s state which inevitably causes time-varying changes in SSAEF [19, 45–49]. Accordingly, some studies have been reported to extract ASSR using independent component analysis (ICA) or other advanced signal processing tools [50]. The ICA-based approach premises either a supergaussian or subgaussian probability distribution for signal extraction and assumes no more than one Gaussian source existing in the signal [51]. The assumption of supergaussian or subgaussian for ICA is inappropriate to interpret the sinusoidal-like nature of ASSR. This study applied CEEMD to extract multi-scale oscillatory activities in MEG data. By projecting the decomposed IMFs on multi-channel MEG data, the spatial distribution of each IMF can be displayed as a spatial map. The spatial information can be further incorporated with the frequency information of IMF to facilitate the selection of SSAEF-related IMFs. The proposed CEEMD-based approach is promising which preserves higher current strengths of neural sources than conventional filtering method (see Tables 2, 3) and more accurate source estimation results (see Figure 6).

It has been understood that noise assisted data analysis (NADA) is an effective way to eliminate mode mixing in EMD-based approaches. By adding noise to perturb the signal in a true solution neighborhood, the ensemble mean of all possible solution can approach the true solution. The NADA inspired Wu et al. [52] to develop EEMD, which repeatedly performs the sifting process on a noise-added signal for a mass of trials. However, the reduction of residual noise in EEMD is time-consuming which requires a large amount of trials for average. The CEEMD [25] elaborately eliminates this problem by adding pairs of positive and negative white noises. Residual noise in extracted IMF can be reduced by taking the mean of theses complementary IMFs as shown in Figure 4.

The benefit of CEEMD in noise removal had demonstrated from our source estimation results. In Figure 6c, the 2D magnetic field reconstructed by CEEMD-based approach shows clear foci at right and left temporal areas. The estimated neural sources were located in the right and left superior temporal cortex (marked by blue arrows). In contrast to the estimated neural sources shown in Figure 6d, unexpected activation in 2D magnetic fields was observed (dashed ellipse). These unwanted neural activities (marked by red arrow in the coronal view of Figure 6d) deteriorated SNR of SSAEF which resulted in lower neural activities in auditory cortex (see Tables 2, 3).

The carrier frequency of auditory stimulus in our study was below patients’ tinnitus frequencies and set at 1,000 Hz. Most of the participants, including normal subjects and tinnitus patients, had the tendency of right-hemisphere dominance (LI > 0) (see Figure 7), responding to 37 Hz steady-state auditory stimulus. The observation of right-hemisphere dominance echoed previous study in healthy subjects [16]. It might be coherent with the hypothesis [53] that the left-hemisphere auditory cortex dominantly processes fast temporal changes whereas the right-hemisphere auditory cortex dominantly processes the spectral information of the sound. Compared to previous studies [42], carrier frequencies of auditory stimuli were set at patients’ tinnitus frequencies. They found the dipole of ASSR was located contralateral to the simulation ear and was positively correlated with subject’s rating of tinnitus intensity. The difference between [16] and [21] might be owing to the carrier frequencies of the two studies were different. In this study, we intend to compare the efficacy of CEEMD-based approach in SSAEF extraction. Therefore, the carrier frequency setup was chosen at 1,000 Hz following Ross et al.’s study [16]. Since the tinnitus frequencies are usually distinct from different tinnitus individuals, the use of same carrier frequency for both healthy and tinnitus groups enables SSAEFs from the two groups can be compared.

## Conclusions

The present CEEMD-based approach features an IMF spatial map creation process and a spatial template matching step for extracting SSAEF-related activities in multi-channel MEG signals. The validity of the proposed method has been examined and its superiority to conventional filtering method has been demonstrated. In this study, we utilized auditory stimulus with carrier frequency lower to patient’s tinnitus frequency and found the phenomenon of right-hemisphere dominance in both normal and tinnitus groups. The estimated neural strengths in tinnitus group were larger than those in normal group.
